# Inhibition of cyclo-oxygenase 2 reduces tumor metastasis and inflammatory signaling during blockade of vascular endothelial growth factor

**DOI:** 10.1186/2045-824X-3-22

**Published:** 2011-10-06

**Authors:** Jason C Fisher, Jeffrey W Gander, Mary Jo Haley, Sonia L Hernandez, Jianzhong Huang, Yan-Jung Chang, Tessa B Johung, Paolo Guarnieri, Kathleen O'Toole, Darrell J Yamashiro, Jessica J Kandel

**Affiliations:** 1 Department of Surgery, Cincinnati Children's Hospital and Medical Center, 3333 Burnet Ave, Cincinnati, 45229-3039, USA; 2 Department of Surgery, College of Physicians and Surgeons of Columbia University, 630 W. 168th St., New York, New York 10032, USA; 3 Department of Pathology, College of Physicians and Surgeons of Columbia University, 630 W. 168th St., New York, New York 10032, USA; 4 Department of Pediatrics, College of Physicians and Surgeons of Columbia University, 630 W. 168th St., New York, New York 10032, USA; 5 Center for Computational Biology and Bioinformatics, College of Physicians and Surgeons of Columbia University, 1130 St. Nicholas Ave, New York, New York 10032, USA

**Keywords:** COX-2, angiogenesis, metastasis, VEGF, inflammation, macrophage

## Abstract

Vascular endothelial growth factor (VEGF) blockade is an effective therapy for human cancer, yet virtually all neoplasms resume primary tumor growth or metastasize during therapy. Mechanisms of progression have been proposed to include genes that control vascular remodeling and are elicited by hypoperfusion, such as the inducible enzyme cyclooxygenase-2 (COX-2). We have previously shown that COX-2 inhibition by the celecoxib analog SC236 attenuates perivascular stromal cell recruitment and tumor growth. We therefore examined the effect of combined SC236 and VEGF blockade, using the metastasizing orthotopic SKNEP1 model of pediatric cancer. Combined treatment perturbed tumor vessel remodeling and macrophage recruitment, but did not further limit primary tumor growth as compared to VEGF blockade alone. However, combining SC236 and VEGF inhibition significantly reduced the incidence of lung metastasis, suggesting a distinct effect on prometastatic mechanisms. We found that SC236 limited tumor cell viability and migration *in vitro*, with effects enhanced by hypoxia, but did not change tumor proliferation or matrix metalloproteinase expression *in vivo*. Gene set expression analysis (GSEA) indicated that the addition of SC236 to VEGF inhibition significantly reduced expression of gene sets linked to macrophage mobilization. Perivascular recruitment of macrophages induced by VEGF blockade was disrupted in tumors treated with combined VEGF- and COX-2-inhibition. Collectively, these findings suggest that during VEGF blockade COX-2 may restrict metastasis by limiting both prometastatic behaviors in individual tumor cells and mobilization of macrophages to the tumor vasculature.

## Background

Agents that inhibit vascular endothelial growth factor (VEGF) signaling are increasingly incorporated into treatment regimens for metastatic human cancer, yet the overall benefit of this treatment strategy has been relatively modest [1,2]. Both clinical and experimental studies indicate that many or most malignancies will ultimately progress if VEGF blockade is sustained, and that progression may involve both progressive primary tumor growth and enhanced metastasis. The mechanisms for acquired resistance to this treatment approach are thus of great interest, but are still emerging. We previously found that VEGF inhibition significantly reduced primary tumor growth and the incidence of spontaneous lung metastasis in the orthotopic renal SKNEP1 tumor model over a six week treatment period, and regressed established metastases in late-stage tumors [3,4]. Recent findings, however, indicate that disruption of VEGF signaling and consequent tumor hypoxia may ultimately promote invasion and metastasis in several tumor models [5,6], overcoming the initial anti-metastatic effects of limiting angiogenesis. Prior studies suggest that hypoxia-regulated and proinflammatory genes expressed by tumor cells and stroma, such as *COX-2*, can promote the establishment of metastatic deposits in the lung. For example, Massague and coworkers previously found that *COX-2 *and other genes involved in vascular remodeling, identified as components in a "lung metastasis gene signature", functioned collectively to promote metastasis in a breast cancer model [7,8]. More broadly, much recent data supports a role for systemic inflammation in the promotion of metastasis in general [9], including dissemination to the lung [10]. For example, mice genetically prone to autoimmune arthritis are significantly more prone to develop lung metastasis than nonarthritic controls [11]. Recruitment of COX-2-expressing macrophages can create an inflammatory proangiogenic environment that strongly promotes tumor growth [12].

Prior work demonstrates that the celecoxib analog SC236 can reduce spontaneous and experimental metastasis, although it is not clear whether this is due to effects on individual tumor cells, on the primary tumor (e.g. angiogenesis), or on the host environment [13]. Thus, it is not known whether the addition of SC236 would limit spontaneous lung metastasis in hypoperfused tumors as occurs during VEGF blockade, when primary tumor angiogenesis is restricted but other prometastatic mechanisms may be active.

In previous studies in the SKNEP1 model, we found that treatment with SC236 perturbed tumor angiogenesis and reduced tumor weights [14]. SC236 treatment resulted in the formation of erratic, segmentally dilated tumor vessels, marked by a decrease in early pericytes and a marked reduction in differentiated vascular mural cells (VMC). These alterations in vessel structure observed in SC236-treated xenografts differed strikingly from those previously found in this same model during administration of blocking anti-VEGF antibody [15]. The potential importance of remodeling is supported by previous work indicating that periendothelial mural cells serve to protect endothelium during VEGF withdrawal [16], and by experiments showing that targeting VMC (e.g. by blockade of platelet-derived growth factor B signaling) in combination with VEGF inhibition enhances anti-angiogenesis [17,18]. It is not known whether SC236 attenuates acquisition or function of perivascular stromal cells during VEGF inhibition, thus potentially reducing vessel stability.

In these studies, we used the SKNEP1 model to ask whether COX-2 inhibition might increase the efficacy of VEGF blockade, either by attenuating vessel remodeling, reducing primary tumor blood flow and tumor growth, or by improving control of metastasis. We asked whether restriction of metastasis could be mediated by direct effects of SC236 on SKNEP1 tumor cells, and whether these were altered in hypoxia, by studying tumor proliferation *in vivo *during SC236 and anti-VEGF treatment, and metabolic and invasive properties of tumor cells *in vitro*. We asked whether tumor expression of a subset of a lung metastasis signature containing COX-2 [7], and more broadly if expression of genes involved in macrophage recruitment, changed during SC236 treatment. Because these mechanisms may collectively contribute to tumor progression in conditions of reduced perfusion, understanding the effects of COX-2 inhibitors during VEGF blockade may hold promise for improving the usefulness of this treatment strategy.

## Materials and methods

### Cell line

The cell line SKNEP-1 (obtained from the American Type Culture Collection, Manassas, VA) was maintained in culture in 75-cm2 flasks with McCoy's 5A medium (Mediatech, Fisher Scientific, Springfield, NJ). Medium was supplemented with 10% fetal bovine serum and 1% penicillin/streptomycin (Life Technologies, Grand Island, NY). Cells were grown at 37°C in 5% CO2 until confluent, harvested, counted with trypan blue staining, washed, and resuspended in sterile PBS (Life Technologies) at a concentration of 10^7 ^per mL.

### Animal model

The Institutional Animal Care and Use Committee of Columbia University approved all experiments. Four- to 6-week-old female NCR nude mice (National Cancer Institute at Frederick, Frederick, MD) were housed in a barrier facility and acclimated to 12-hour light/dark cycles for at least 24 hours before experimental use.

### Tumor implantation

Mice (n = 62) were anesthetized with intraperitoneal ketamine (50 mg/kg) and xylazine (5 mg/kg). The left flank was prepared in a sterile fashion, and an incision was made, exposing the left kidney. An inoculum of 10^6 ^SKNEP1 tumor cells in 0.1 ml of PBS was injected into the renal parenchyma using a 25-gauge needle. The flank musculature was closed with a single 4-0 Polysorb suture (U.S. Surgical, Norwalk, CT), and the skin incision was closed with staples.

### Administration of SC236 and anti-VEGF antibody

One week after tumor implantation, animals were divided into four cohorts. Treated mice received either (1) SC236 (N = 17; Pfizer, Groton, CT) added to drinking water at a concentration of 30 μg/mL and changed thrice per week as previously described [14]; (2) the humanized monoclonal anti-VEGF antibody bevacizumab (N = 17; BV; Genentech, South San Francisco, CA) injected intraperitoneally at 10 mg/kg biweekly beginning at week 3; or (3) combined agents (N = 15). Control mice (N = 13) received standard drinking water and were injected with carrier vehicle on the same schedule. This concentration of SC236 is equivalent to ~6 mg/kg/d (anticipated SC236 plasma level 5 μg/ml). Plasma levels of SC-236 were previously confirmed in our model by high-performance liquid chromatography (HPLC) [14].

### Harvesting of specimens and determination of metastases

At sacrifice, tumors and contralateral kidneys were removed, weighed, and then preserved in 4% paraformaldehyde for immunohistochemistry. Portions of tumor were flash frozen in liquid nitrogen and stored at -80°C. Both lungs were fixed in 10% formalin for histology. Slides of paraffin-embedded lung tissue were stained by routine H&E methods and examined by a surgical pathologist to determine the presence or absence of metastases, using three levels from each lung.

### Immunohistochemistry

Endothelial cell immunofluorescent staining was done using a rat anti-mouse anti-platelet-endothelial cell adhesion molecule-1 (PECAM-1) monoclonal antibody (MAP0032, Angioproteomie, Boston, MA). Vascular mural cells (VMC) were visualized using a rabbit anti-human α-smooth muscle actin (αSMA) antibody (RB-9010, Lab Vision/Neomarkers, Fremont, CA). Macrophages were visualized using an anti-murine F4/80 antibody (Abcam, AB6640) and an anti-arginase antibody (Santa Cruz Biotech, SC-20150). All microscopy was done using a Nikon Eclipse E600 apparatus.

### Quantification of proliferation

*In vivo *tumor proliferation (N = 3 for all groups) was examined by immunofluorescent staining for anti-phospho-histone H3 (Upstate, Inc., Lake Placid, NY), and quantified by computer-assisted image analysis (23-30 fields per group examined).

### MMP-2 and -9 zymography

Tumor extracts (N = 3 for each group) were normalized for protein content, mixed with nonreducing sample buffer, and electrophoresed in 10% polyacrylamide gels copolymerized with 1 mg/mL of gelatin. After electrophoresis, gels were washed twice for 15 min in 2.5% Triton X-100, rinsed with water, and incubated overnight in activation buffer (50 mM/L Tris-HCl, pH 7.5, 5 mM/L CaCl2) at 37°C. Digested gelatin bands were visualized by staining with 0.1% Coomassie blue R-250 in 40% methanol/20% acetic acid. Pooled MMPs were used as a positive control (US Biological). Bands were optically scanned for quantification.

### MTT Assay

2 × 10^5 ^SKNEP cells/well were incubated on a 24-well plate (BD Labware, Franklin Lakes, NJ) for 24 hours. Media was then replaced with control media or media with 25 or 50 uM SC-236 (Pfizer, New York, NY) and incubated for 24 or 96 hours at 37°C in either normoxia (21% oxygen) or hypoxia (2.3% oxygen). After the incubation period, media was aspirated and replaced with serum-free media and MTT labeling reagent (Roche, Indianapolis, IN). After 24 hours, 600 uL isopropanol was added to each well and the resultant solution read in a Life Science UV spectrophotometer (Beckman, Fullerton, CA) at wavelengths of 550 and 690 nm. All samples were plated in triplicate and experiments were repeated 3 times. Assays were repeated with added bevacizumab (100 ng/ml).

### Invasion Assay

SKNEP cells were serum-starved for 24 hours. 5 uM Cell Tracker Green (Invitrogen) was added to the cells and incubated for 45 min. Media was then aspirated and cells incubated in serum-free McCoy's solution for an additional 45 min. 8 × 10^5 ^cells/well were plated in 24-well, 8 um pore, Matrigel-coated Tumor Invasion System plates (BD Biosciences) with serum-free media containing 0 (control), 25, or 50 uM SC-236. The same media with 15% FBS was added to the lower well, and the plate incubated for 24 hours in either normoxia or hypoxia (as above). Invading cells were quantified using a Cytofluor series 4000 Fluorescence multi-well plate reader (Applied Biosystems, Foster City, CA) at an excitation wavelength of 485 nm and an emission wavelength of 530 nm. All samples were plated in triplicate and experiments were repeated 3 times.

### Apoptosis

10^5 ^tumor cells per well were plated on a 96-well Microtest Tissue Culture Plate (BD Labware). Either media or media with 25 or 50 uM SC-236 was added, and allowed to incubate in either normoxia or hypoxia for 20 hours. Apoptosis was analyzed using the Cell Death Detection Elisa kit (Roche, Indianapolis, IN). Briefly, media was aspirated, and cells lysed. After a 30 minute incubation period, the solution was centrifuged at 1250 rpm for 10 minutes and the supernatant added to a streptavidin-coated plate. A mixture of anti-histone-biotin and anti-DNA-peroxidase solution was added to each well and mixed for 2 hours. Substrate was then added, and color intensity read using a VersaMax tunable microplate reader (Molecular Devices, Sunnyvale, CA) at a wavelength of 405 nm. Camptothecin was used as a positive control for apoptosis.

### Real-time PCR for lung metastasis signature genes

Total RNA was isolated from xenograft tumors (N = 4 each), using an Ambion ToTally RNA Extraction kit (Ambion, Austin, TX). Real-time PCR was performed using Taqman probes and an ABI 7300 (Applied Biosystems). Normalization of genes of interest was done utilizing the geNorm method described by Vandesompele et al [19]. Six human housekeeping genes (ACTB, HMBS, UBC, GAPD, HPRT, PPIA) were used as reference controls.

### Microarrays and gene set expression analysis

HG-U133A GeneChips (Affymetrix, Santa Clara, CA) were used to investigate gene expression in xenografts. cRNA probes were synthesized as recommended by Affymetrix. Briefly, total RNA was isolated in two steps using ToTALLY RNA Total RNA isolation kit (Ambion, Austin, TX) followed by RNeasy (Qiagen, Valencia, CA) purification. Double-stranded cDNA was generated from 5 μg of total RNA using a polydT oligonucleotide that contained a T7 RNA polymerase initiation site and the Superscript Choice System kit (Invitrogen, Carlsbad, CA). Biotinylated cRNA was generated by *in vitro *transcription using the Bio Array High Yield RNA Transcript Labeling System (Enzo, Farmingdale, NY). cRNA was purified using RNeasy and fragmented according to the Affymetrix protocol, and 15 μg of biotinylated cRNA hybridized to HGU133A microarrays (Affymetrix). Raw CEL files were processed using Bioconductor packages in an R environment http://www.bioconductor.org) [20]. Briefly, quality controls were performed by inspecting Affymetrix^® ^metrics using the simpleaffy package. Probe level signals were then background-corrected, normalized, and summarized using the GC-RMA function. Differential gene expression was computed using an Empirical Bayesian model implemented in the Limma package (Additional File 1 Table S1). To determine whether the addition of SC236 broadly changed inflammation-related pathways in BV-treated tumors, we used Gene Set Expression Analysis and GenePattern tools (GSEA, Broad Institute, Cambridge, MA). The MSigDB gene set database (5,542 total sets) was queried for gene sets containing inflammation-related pathways. From this, we constructed a 67 gene set matrix. GSEA was used to assess expression of gene sets in this matrix in BV- and BV+SC236-treated samples (N = 3, 4 respectively). To compute gene enrichment, we permuted by gene (as recommended for small sample sizes) 5000 times. We then computed odds ratios for the genesets identified as being significant by GSEA, defined as ratio of odds for the hits before and after the leading edge. In particular, for each geneset we computed the following:

oddsRatios =[(Hits/Misses) BeforeTheLeadingEdge]/[(Hits/Misses) AfterTheLeadingEdge]

We used the odds ratios to additionally filter the results, as nominal p-value is not informative in this setting, and the NES has a bias towards bigger genesets.

### Enzyme-linked immunosorbent assay (ELISA)

ELISA for tumor-derived (human) IL4 was performed using the Human IL-4 Quantikine HS ELISA Kit HS400 (R&D Systems). All assays were performed in duplicate, and then repeated twice. Results of repeated assays were expressed as summary means ± standard error (square root of interassay variance) [21].

### Statistical analysis

Tumor weights were compared by Kruskal-Wallis analysis. The presence or absence of lung metastases was evaluated by Fisher's exact test. Quantitative results of multiply-repeated MTT, invasion, and ELISA assays were assessed using Comprehensive Meta Analysis software (Biostat, Inc., Englewood, NJ). Differences in gene expression by real-time PCR were compared by Student's T-test.

### Quantification of changes in vasculature

Digital images from immunofluorescence and fluorescein-labeled lectin studies were captured from a Nikon E600 fluorescence microscope with a Spot RT slider digital camera (Diagnostic Instruments, Sterling Heights, MI) and stored as TIFF files. Vessel radius was determined by immunofluorescence for αSMA and analyzed using software Adobe Photoshop 7.0 (Adobe Systems, Mountain View, CA) and Image Processing Toolkit (IPTK 5 IPTK 5, Reindeer Graphics, Raleigh, NC). Briefly, background fluorescence is subtracted (AutoLevelDark), Gaussian filter applied to reduce electronic and background noise (Gaussian Blur, radius = 1.0 pixel), and grayscale levels linearly expanded (Auto Levels). A common threshold level is applied that preserves correct vascular morphology (Threshold). The image is inverted (Invert) and dilated to lessen gaps (Classic Morphology > Dilate), small particles representing background removed (Reject Features), and vessel holes filled in (Fill Holes). The average of the inscribed radius is determined as a measure of vessel radius. 60 images from SK-NEP1-GFP (3 different tumors) and 49 images from SK-NEP1-Ang1* (3 different tumors) were analyzed. To assess MVD and vessel length, quantitative assessment of tumor vessel architecture was performed by computer-assisted digital image analysis as previously described [22]. The fraction of fluorescein-labeled lectin-positive pixels per total field was quantified. Specific changes in vessel architecture are evaluated by quantifying branch points (nodes), end points, and total vessel length. Images are analyzed after application of a common threshold, image inversion, morphological erosion, and skeletonization, again using a combination of Photoshop and Image Processing Toolkit.

## Results and Discussion

### The addition of SC236 to VEGF inhibition does not further restrict tumor size, but does significantly reduce the incidence of metastasis as compared to anti-VEGF treatment alone

**Figure 1:**
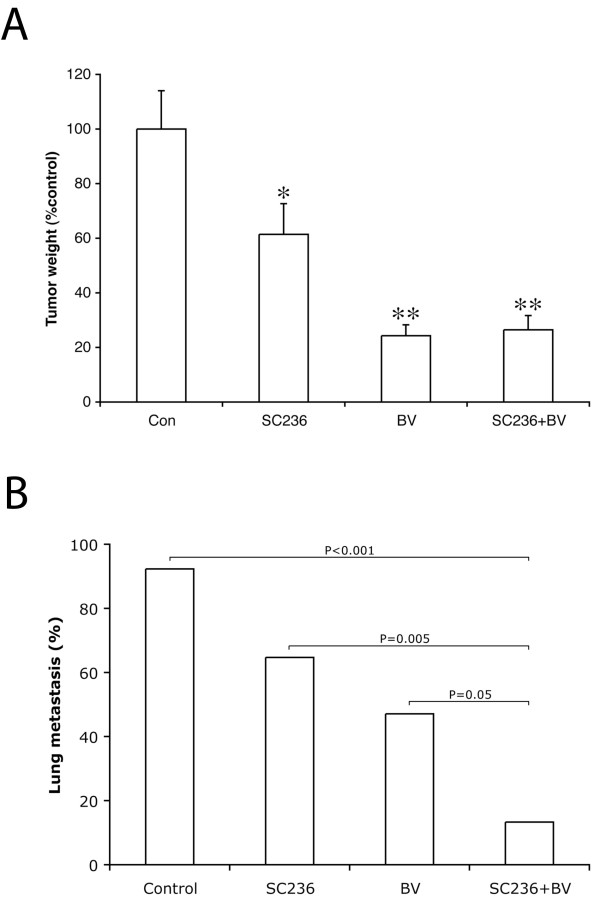
**The addition of SC236 to bevacizumab significantly reduces the incidence of metastasis as compared to VEGF inhibition alone**. **(A) **As compared to controls, SC236 treatment reduced tumor weight by 39% (*P = *0.042), whereas BV-treated tumors were reduced by 76% (*P = *0.0002). The addition of SC236 to BV treatment did not further decrease primary tumor weight (74% reduction from controls, *P = *NS as compared to BV alone). Tumor weights are expressed as percentage of control tumors, and Kruskal-Wallis analysis used to determine significance. **(B) **The incidence of metastasis was determined by examining 3 levels from the right lung. 12/13 or 92% of control animals had detectable metastases, whereas 11/17 (65%) of SC236 treated animals had lung deposits (*P = *NS). The incidence of metastasis in BV-treated animals was significantly reduced as compared to controls (8/17 animals, 47%, *P = *0.011). Combining SC236 with BV treatment further significantly reduced metastasis (2/15 animals, 13%, *P = *0.0001 as compared to controls and *P = *0.046 as compared to BV treatment alone).

As compared to controls, SC236 treatment reduced tumor weight by 39% (*P = *0.042, Kruskal-Wallis analysis; Figure 1A), whereas tumors treated with the anti-VEGF antibody bevacizumab (BV) were reduced by 76% (*P = *0.0002). The addition of SC236 to BV treatment did not further decrease primary tumor weight (74% reduction from controls, *P = *NS as compared to BV alone). The incidence of lung metastasis was determined by a surgical pathologist blinded as to treatment group, examining 3 levels from the entire right lung (Figure 1B). Nearly all of the control animals had detectable metastases (12/13 mice, 92%). 65% (11/17) of SC236 treated animals had lung metastases, but this reduction was not statistically significant. The incidence of metastasis was, however, significantly reduced in BV-treated mice to 47% (8/17 mice), which was significantly different when compared to controls (*P = *0.011). Strikingly, combining SC236 with BV treatment caused a further reduction in metastasis with only 13% (2/15) of mice having lung metastases. This effect was statistically significant when compared not only to control tumors (*P = *0.0001), but also when compared to BV treatment alone (*P = *0.046). We examined lung metastases histologically using H&E staining (Additional File 2 Figure S1). Metastases in combination-treated mouse lungs appeared smaller than in control or single-agent treated animals, although the low incidence of metastasis in this group (2/15) prevented quantitation of this size reduction. Thus, our data demonstrates that SC236 when combined with VEGF blockade can markedly reduce distant metastasis, although it does not alter primary SKNEP1 tumor size, suggesting a differential effect on this aspect of tumor progression.

### The combination of SC236 with VEGF inhibition alters vascular assembly as compared to either treatment alone

**Figure 2:**
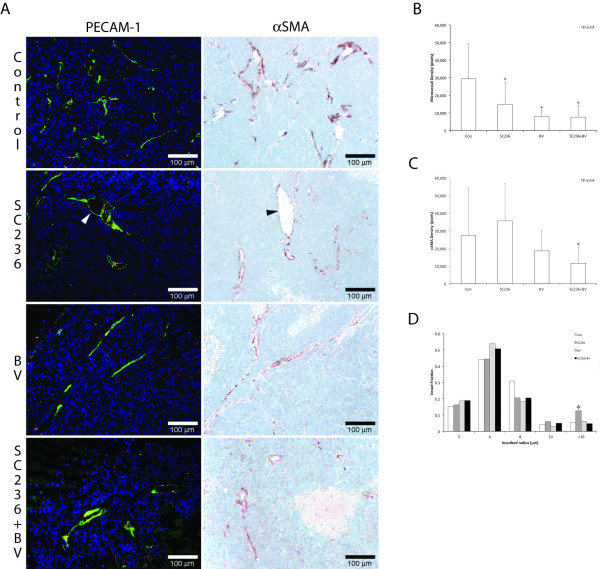
**The combination of SC236 with VEGF inhibition alters vascular assembly as compared to either treatment alone**. We performed immunohistochemistry for platelet endothelial cell adhesion molecule-1 to assess changes in endothelial vessels (PECAM; Figure 2, left column). Control tumors displayed abundant PECAM(+) neovasculature, with multiple fine branches (arrows). Morphologically, both SC236-treated and BV-treated tumors displayed reduction of branching. In tumors treated with both BV and SC236, surviving endothelial vessels were discontinuous, with irregularly spaced, moderately dilated segments, and near ablation of branches. Mean vascular density was decreased in all treated groups as compared to controls (Figure 2B). To examine differentiated VMC, we immunostained for α-smooth muscle actin (αSMA, Figure 2 right column). Quantitation of αSMA immunopositive vasculature demonstrated significant reduction only in BV+SC236 treated tumors as compared to controls (Figure 2C), potentially consistent with a combined effect on microvessel density and disrupted mural cell coverage of vessels as previously reported [14]. PECAM- and αSMA-immunostaining also suggested that SC236-treated xenografts were marked by erratically dilated larger vessels (arrowheads, Figure 2A). To quantify this morphologic change, we measured shifts in distribution of the inscribed radius of mural cell-covered vasculature (Figure 2D, using a previously described method [22]). SC236-treated tumor vasculature demonstrated a significant increase in the vessels with inscribed radius > 10 μm.

To assess changes in the endothelial component of vessels, we performed immunohistochemistry for platelet endothelial cell adhesion molecule-1 (PECAM; Figure 2, left column). Control tumors displayed abundant PECAM(+) neovasculature, with multiple fine branches (arrows). Morphologically, both SC236-treated and BV-treated tumors displayed reduction of branching. In tumors treated with both BV and SC236, surviving endothelial vessels were discontinuous, with irregularly spaced, moderately dilated segments, and near ablation of branches. Mean vascular density was decreased in all treated groups as compared to controls (Figure 2B, p < 0.05). To examine differentiated VMC, we immunostained for α-smooth muscle actin (αSMA, Figure 2, right column). Quantitation of αSMA immunopositive vasculature demonstrated significant reduction in BV+SC236 treated tumors as compared to controls (Figure 2C), potentially consistent with a combined effect on microvessel density and disrupted mural cell coverage of vessels as previously reported [14]. Thus, the addition of SC236 to BV treatment may have increased discontinuity in tumor vessel networks, with relative attenuation of VMC recruitment.

PECAM- and αSMA-immunostaining also suggested that SC236-treated xenografts were marked by erratically dilated larger vessels. To quantify this morphologic change, we measured shifts in distribution of the inscribed radius of mural cell-covered vasculature (Figure 2D, using a previously described method [22]). SC236-treated tumor vasculature demonstrated a significant increase in the vessels with inscribed radius > 10 μm.

### SC236 affects proliferation and invasive properties of SKNEP1 tumors *in vivo *and *in vitro*

**Figure 3:**
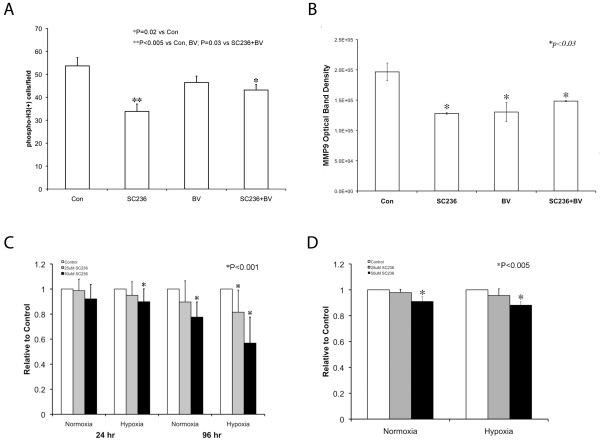
**SC236 affects proliferation and invasive properties of SKNEP1 tumors *in vivo *and *in vitro***. **(A) **Quantification of phosphohistone H3 (pH3) (N = 3; 23-30 sections studied, each condition) demonstrated that SC236 reduced proliferation *in vivo *as compared to both control and BV-treated tumors (33.8 ± 3.2 cells/hpf vs. 53.6 ± 3.6 and 46.4 ± 2.8 cells/hpf, respectively; P < 0.005, both). Combined SC236+BV treatment also reduced proliferation versus controls (43.1 ± 2.4 cells/hpf, P < 0.02). Thus, SC236 restriction of proliferation persisted during VEGF blockade, but was not altered. **(B) **We assessed MMP-2 and MMP-9 activation, which can promote lung metastasis and interacts with VEGF and COX-2 status, using gelatin zymography [23-26] in each treatment group (N = 3 each). MMP-2 activity was unchanged (not shown). MMP-9 activity was significantly reduced in each treatment group as compared to controls (versus SC236 group, 35%; in BV, 34%, SC36+BV, 25%; P < 0.03, each). Thus, combined SC236 and BV did not further reduce MMP9 activity. **(C) **We used the MTT assay to determine whether hypoxia, SC236 concentration, or duration of exposure would reduce SKNEP1 tumor cell viability. Both hypoxia and increasing concentration suppressed activity measured by MTT, with effects enhanced by increasing duration (96 h). These results support an increased effect of SC236 in hypoxic conditions, as may occur during VEGF blockade. **(D)**. We assessed SC236 effect on tumor cell invasion through basement membrane (Matrigel). Invasion was reduced at 50 μM SC236 by 7% in normoxia and 14% in hypoxia (P < 0.005, each).

We asked whether SC236 caused changes in metastasis-related tumor cell behaviors under conditions imposed by VEGF blockade, including proliferation, activation of matrix metalloproteases (MMP) -2 and -9 and ability to invade basement membrane. **(A) **COX-2 is known to modulate tumor cell proliferation, and we previously found that SC236 decreased tumor proliferation *in vivo*. However, it is not known whether this effect would be altered in hypoxia, which might enhance sensitivity to COX-2 or (conversely) stimulate other pathways that promote proliferation. We quantitated phosphohistone H3 (pH3), using image analysis of immunofluorescent staining (N = 3 and 23-30 sections studied, each condition). SC236 reduced pH3 immunopositive cells *in vivo *(Figure 3A), as compared to both control and BV-treated tumors (33.8 ± 3.2 cells/hpf vs. 53.6 ± 3.6 and 46.4 ± 2.8 cells/hpf, respectively; P < 0.005, both). Combined SC236 and BV treatment also reduced proliferation in comparison to controls (43.1 ± 2.4 cells/hpf, P < 0.02). Thus, SC236 restriction of proliferation persisted during VEGF blockade, but was not significantly altered. **(B) **Activation of matrix metalloproteinases has been linked to metastasis of primary tumors to the lung [23,24], and can be modulated by VEGF and COX-2 signaling [25,26]. Using gelatin zymography, we examined MMP-2 and MMP-9 in tumor samples, and quantified activity by comparing optical band density in each treatment group (Figure 3B, N = 3 each). MMP-2 activity was unchanged in each condition (not shown). MMP-9 activity was significantly reduced in each treatment group as compared to controls (versus SC236 group, 35%; in BV, 34%, BV+SC36, 25%; P < 0.03, each). Thus, combined SC236 and BV did not further reduce MMP9 activity. **(C) **We used the MTT assay to determine whether hypoxia, SC236 concentration, or duration of exposure would reduce viability in SKNEP1 tumor cells. We have previously reported that 30 μg/mL SC236 in drinking water results in serum concentrations between 25 and 50 μM/mL [14]; therefore, we tested effects at these two concentration levels. We found that both hypoxia and increasing concentration suppressed activity measured by MTT, with this effect enhanced at increased duration (96 h). For example, at 24 h significant reduction as compared to controls (9.9%, P < 0.001) was found only at the higher concentration (50 μM) and in hypoxia, whereas at 96 h significant reduction was found in normoxia at the 50 μM concentration (8%), and in hypoxia at both 25 and 50 uM (16% and 44%, respectively; P < 0.001, all). These results support an increased effect of SC236 in hypoxic conditions, as may occur in tumor regions during VEGF blockade. We repeated these studies in the presence of BV, and found no effect. **(D) **We assessed the ability of SC236 to restrict tumor cell invasion through basement membrane (Matrigel), since intravasation is a key step in metastasis. Invasion was significantly reduced only at the 50 μM concentration level, by 7% in normoxia and 14% in hypoxia (P < 0.005, each), consistent with a modest restriction by SC236. In summary, SC236 reduces metastatic behaviors in tumor cells: proliferation *in vivo *and viability and migration through basement membrane *in vitro*, with the latter two effects enhanced by hypoxia.

### SC236 does not increase apoptosis in SKNEP1 cells in normoxia or hypoxia

COX-2 signaling can interfere with apoptotic mechanisms in some cell types and in some settings. Therefore, we examined the effect of SC236 on SKNEP1 cell apoptosis *in vitro*. No increase in apoptosis was found at either concentration of SC236, and no effect of hypoxia was detected (not shown). Thus, increasing apoptosis may not contribute to restriction of metastasis in this system.

### The addition of SC236 does not alter expression of lung metastasis signature genes during VEGF blockade

We detected expression of 7 genes (COL6A1, CSF2RA, CXCR4, KRT81, MATN2, SPARC, TNC) included in the lung metastasis gene signature described by Minn et al. [8] in tumor extracts from each group (N = 6, controls, SC236-treated, BV-treated; N = 5, BV+SC236 treated; Additional File 3 Figure S2). Comparison was performed by real-time PCR. No significant differences between BV and SC236+BV treated tumors were found, suggesting that SC236 did not reduce lung metastasis by suppressing expression of these genes.

### The addition of SC236 alters expression of gene sets representing macrophage-related inflammatory pathways in BV-treated tumors

COX-2 activity broadly influences inflammation, which appear increasingly implicated in tumor development, progression, and metastasis [9]. It is possible that SC236 improves control of metastasis in BV-treated tumors in our model by reducing expression of inflammation-related genes that act in concert. We therefore used gene set expression analysis (GSEA) and a matrix of 67 gene sets representing pathways linked to macrophage recruitment selected from the Molecular Signatures Database (MSigDB, Broad Institute, Cambridge, MA) to explore this possibility in Affymetrix HGU133A microarray datasets from xenografts (N = 3, 4 respectively, for BV, and BV+SC236 groups). 20 gene sets were significantly negatively enriched (repressed) in combination in SC236+BV treated as compared to BV-treated tumors (Table 1). No positively enriched gene set had an odds ratio > 2.0, whereas the top 5 negatively enriched gene sets were significant both by GSEA normalized enrichment score and had odds ratios > 2. Leading edge analysis of these sets suggested involvement of cytokines, with interleukin-4 (IL40 the most significantly suppressed gene in the leading edge analysis. IL4 plays a key role in recruiting and activating tumor-associated macrophages [27] and can be suppressed by COX-2 inhibition in inflammatory conditions [28]. Therefore, we examined expression of IL4 protein by ELISA in BV-treated versus SC236+BV-treated tumors, and found a 60% reduction in combined-treatment tumors (3.1 ± 1.7 pg/ml, BV treated; 1.2 ± 0.3 pg/ml, SC236+BV-treated, *P *= 0.056).

**Table 1:** The addition of SC236 alters expression of gene sets associated with macrophage recruitment pathways in BV-treated tumors

POSITIVELY ENRICHED GENE SETS						
**NAME**	**ES**	**NES**	**NOM p-val**	**FDR** **q-val**	**FWER p-val**	**oddRatios**

DNA_METABOLIC_PROCESS	0.3612	1.8297	0.0000	0.0074	0.0134	1.9304
APOPTOSIS_KEGG	0.3195	1.2502	0.1253	0.2551	0.6220	1.9831
SOLUBLE_FRACTION	0.2035	0.9724	0.5341	0.8663	0.9962	1.5214
NEGATIVE_REGULATION_OF_PROGRAMMED_CELL_DEATH	0.1657	0.7811	0.9662	0.8935	1.0000	1.2876
						

**NEGATIVELY ENRICHED GENE SETS**						

**NAME**	**ES**	**NES**	**NOM p-val**	**FDR** **q-val**	**FWER p-val**	**oddRatios**

***HSA04060_CYTOKINE_CYTOKINE_RECEPTOR_INTERACTION***	***-0.5365***	***-2.2777***	***0.0000***	***0.0000***	***0.0000***	***3.5618***
***CYTOKINE_ACTIVITY***	***-0.5464***	***-2.1044***	***0.0000***	***0.0000***	***0.0000***	***3.3868***
IMMUNE_RESPONSE	-0.4609	-1.9351	0.0000	0.0007	0.0018	2.3685
DEFENSE_RESPONSE	-0.4529	-1.9243	0.0000	0.0006	0.0022	2.2449
***INFLAMMATORY_RESPONSE_PATHWAY***	***-0.6176***	***-1.9127***	***0.0009***	***0.0006***	***0.0026***	***3.4287***
REGULATION_OF_MULTICELLULAR_ORGANISMAL_PROCESS	-0.4773	-1.9076	0.0000	0.0005	0.0026	2.0887
CYTOKINE_PRODUCTION	-0.5188	-1.8253	0.0005	0.0017	0.0108	3.8765
HSA04514_CELL_ADHESION_MOLECULES	-0.4568	-1.7974	0.0002	0.0020	0.0144	1.7020
IMMUNE_SYSTEM_PROCESS	-0.4064	-1.7650	0.0000	0.0029	0.0230	1.8520
RESPONSE_TO_EXTERNAL_STIMULUS	-0.4064	-1.7482	0.0000	0.0032	0.0286	2.0471
INFLAMMATORY_RESPONSE	-0.4473	-1.7450	0.0000	0.0031	0.0300	2.1490
POSITIVE_REGULATION_OF_MULTICELLULAR_ORGANISMAL_PROCESS	-0.4977	-1.7303	0.0016	0.0035	0.0374	1.5006
RECEPTOR_BINDING	-0.3842	-1.6798	0.0000	0.0058	0.0648	1.7643
RESPONSE_TO_WOUNDING	-0.4071	-1.6678	0.0000	0.0064	0.0766	2.1108
IMMUNE_EFFECTOR_PROCESS	-0.5187	-1.6357	0.0081	0.0082	0.1052	2.5265
LYMPHOCYTE_ACTIVATION	-0.4637	-1.6207	0.0046	0.0090	0.1222	1.4574
CELL_ACTIVATION	-0.4436	-1.6042	0.0048	0.0102	0.1460	1.9692
LEUKOCYTE_ACTIVATION	-0.4516	-1.6008	0.0054	0.0100	0.1516	2.0518
ADAPTIVE_IMMUNE_RESPONSE_GO_0002460	-0.5491	-1.5398	0.0294	0.0181	0.2656	2.1055
ADAPTIVE_IMMUNE_RESPONSE	-0.5268	-1.4882	0.0446	0.0280	0.3934	2.3657

To assess the possibility that SC236 improves control of metastasis in the context of VEGF blockade by broadly reducing expression of inflammation-related genes, we used gene set expression analysis (GSEA) and a matrix of 67 gene sets representing pathways linked to macrophage recruitment selected from the Molecular Signatures Database (MSigDB, Broad Institute, Cambridge, MA) to explore this possibility in Affymetrix HGU133A microarray datasets from xenografts (N = 3, 4 respectively, for BV, and BV+SC236 groups). 20 gene sets were significantly negatively enriched (repressed) in combination in SC236+BV treated as compared to BV-treated tumors; no positively enriched gene set had an odds ratio > 2.0 (Table 1). Among the negatively enriched sets, we selected those with odds ratios > 3.0 in the top 5 for further analysis. Thus, it is possible that, in the context of VEGF inhibition, SC236 acts in part by limiting the ability of tumors to promote recruitment of macrophages, key elements in an inflammatory state that facilitates metastasis.

### Macrophage recruitment is altered by BV and SC236 treatment

**Figure 4:**
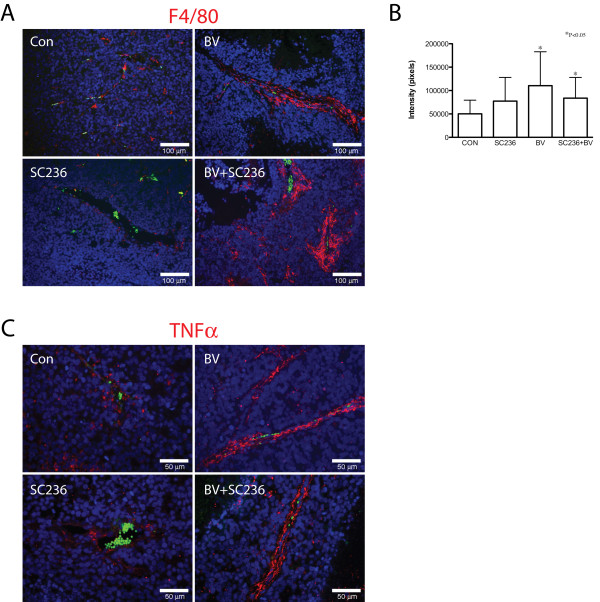
**Macrophage recruitment is altered by BV and SC236 treatment**. Distribution of macrophages was examined using the mouse macrophage marker F4/80 (Figure 4A). Morphologically, F4/80 appeared to be increased in BV- and BV+SC236-treated tumors, with somewhat more irregularity in the latter. Quantified F4/80(+) signal was significantly increased in BV- and SC236+BV-treated groups versus controls (Figure 4B). TNFα-immunopositive cell distribution appeared similar to the patterns for F4/80 signal (Figure 4C). The M2 marker arginase was detected in cells primarily in necrotic regions, which did not change with treatment (not shown).

These results led us to examine distribution of macrophages in treated tumors, using the mouse macrophage marker F4/80 (Figure 4A). Morphologically, there appeared to be increased irregularity of the perivascular F4/80 signal in the combined-treatment group. Quantification of F4/80 immunopositivity demonstrated significant increase in BV and SC236+BV treated groups as compared to controls (Figure 4B). To detect shifts in distribution of polarized macrophages, we performed immunohistochemistry for the M1 marker TNFα (Figure 4C). TNFα-immunopositive cell distribution appeared similar to the patterns for F4/80 signal. Immunohistochemistry for the M2 marker arginase demonstrated immunopositive cells primarily in necrotic areas of tumors; quantification of arginase signal did not change between treatment groups (data not shown).

## Conclusions

While inhibition of VEGF signaling has become an established cancer therapy, it has become evident that its overall impact is relatively modest, with virtually all patients developing progressive disease [5,6]. One proposed mechanism for the limitations of this approach is the alteration in tumor gene expression induced by hypoperfusion. Because it is regulated by hypoxia, a consequence of treatments that restrict blood supply, COX-2 may participate in this compensatory response to VEGF inhibitors. In addition, COX-2 signaling is implicated in multiple processes that contribute to tumor progression, including proliferation, survival, angiogenesis, and invasion. In these studies, we asked whether inhibition of COX-2 by SC236 would enhance the effects of VEGF blockade in the SKNEP1 model, either by perturbing compensatory vascular remodeling and primary tumor perfusion, or by further reducing the incidence of lung metastasis.

SC236 treatment significantly restricted primary tumor weight as compared to controls, as did BV. However, the addition of SC236 to BV treatment did not result in any further reduction, leading us to ask how this combination affected tumor vasculature and consequent tumor viability. As in our prior studies, both agents impaired angiogenesis, with reduction in branching and pruning of smaller vessels [14]. SC236 disrupted vascular assembly, with erratic dilatation of large vessels. Tumors treated with combined agents displayed elements of these two patterns, with preserved VMC coverage of surviving vessels but irregular segmental dilatation and discontinuity. Tumor proliferation was not further reduced *in vivo *from treatment with SC236 alone. Thus, in our model the ability of SC236 to perturb VMC recruitment did not sufficiently destabilize vasculature during VEGF inhibition to further limit tumor growth.

In the SKNEP1 model, inhibition of VEGF reduces the incidence of lung metastasis, and can cause initial regression of established metastases in later-stage tumors [4]. However, SKNEP1 tumors [29] and metastases (our unpublished observation) recur if VEGF inhibition is prolonged. Thus, our system may serve as an example of a tumor type in which metastasis is initially sensitive to VEGF blockade, in contrast to others in which it may actually be increased [5,6]. The addition of SC236 to BV treatment did significantly improve control of metastasis, by reducing incidence. This finding suggests that SC236 may have limited one or more tumor cell behaviors that specifically contribute to establishment of metastasis, such as the ability to transit tumor parenchyma, invade vasculature, or survive as implants in the lung, distinct from effects on primary tumor growth. Consistent with this concept, proliferation in primary tumors was not further restricted in BV+SC236- as compared to SC236-treated xenografts. However, we found that viability of SKNEP1 cells (as assessed by MTT) tended to be restricted by SC236 *in vitro *in hypoxia, particularly if exposure were prolonged or at a higher concentration. It is possible that proliferation is supported in a primary tumor *in vivo *by secreted products of adjacent nonhypoxic regions, since BV+SC236 did not completely devascularize xenografts, or by host cells that contribute to tumor stroma. Invasion of tumor cells into basement membrane *in vitro *was similarly restricted by 50 μM SC236, with possible enhancement of this effect by hypoxia. However, while MMP-9 activity was reduced in both SC236- and BV-treated tumors, there was no further reduction in activity in BV+SC236-treated xenografts, suggesting that decreased invasion may not be mediated by diminished MMP-9. No effect of SC236 on tumor cell apoptosis was detected. Taken together, these findings are consistent with an effect on prometastatic behaviors (viability, invasion of vascular basement membrane) in individual tumor cells.

Because alteration in COX-2 signaling may affect tumor cell expression of other genes that specifically promote lung metastasis, we asked whether SC236 treatment altered expression of a subset of a previously described lung metastasis gene signature that includes COX-2 [7,8]. While expression of these genes was detected in our model system, the addition of SC236 to BV did not reduce expression as compared to BV-treated tumors. More broadly, mobilization of macrophages by an inflammatory tumor microenvironment can also contribute to metastasis [9-11]. Given the ability of SC236 to block COX-2 activity, we asked whether the addition of SC236 to BV treatment limited primary tumor expression of gene sets implicated in macrophage-related pathways. Using gene set expression analysis, we found a shift toward significant suppression of gene sets containing cytokines that mediate such responses. In particular, we detected reduced expression of IL4 mRNA and protein. IL4 plays an established role in activating tumor-associated macrophages, and can be regulated by COX-2 activity. Suppression of this cytokine by COX-2 inhibition in the context of VEGF blockade might therefore contribute to attenuating a prometastatic tumor microenvironment. BV treatment quantitatively increased perivascular macrophage recruitment. The addition of SC236 did not limit this increase, or modulate distribution of cells bearing M1 or M2 markers. Thus, the effect of SC236 on tumor-associated macrophages in the context of VEGF blockade does not appear to include quantitative changes in recruitment.

Thus, our data provides evidence that inhibition of COX-2 may limit metastasis in the setting of VEGF inhibition, distinct from its effect on primary tumor angiogenesis and growth. Restriction of metastasis may result from reduced viability or ability to transit basement membrane in individual tumor cells. In addition, diminished primary tumor angiogenesis may have limited tumor cell access to the circulation, limiting hematogenous dissemination. We detected increased perivascular recruitment of macrophages in BV-treated tumors, and shifts in gene expression related to macrophage function between BV- and BV+SC236-treated tumors. However, we did not detect histologic changes in the distribution of macrophages when SC236 treatment was added to BV. Further investigation of functional effects of COX-2 blockade on interaction of tumor cells and stroma may assist in better understanding of these alterations, and in optimizing the use of anti-VEGF treatments in cancer patients at risk for metastatic progression.

## Competing interests statement

The authors declare that they have no competing interests.

## Authors' contributions

JCF contributed to design, and planned and executed mouse experiments, tissue analyses, gel chromatography, and migration studies. MJH, SLH, and JH performed mouse tumor modeling, immunohistochemistry, and tissue analyses. YJC performed ELISA studies. TBJ performed macrophage studies. PG analyzed raw microarray data. KO analyzed mouse lungs for metastasis. DJY and JJK conceived of the study, supervised its execution, and wrote the manuscript.

All authors read and approved the final manuscript.

## Supplementary Material

Additional file 1 **Table S1**. LimmaAnnotated.xls: Differential gene expression was computed using an Empirical Bayesian model implemented in the Limma package. Briefly, we analyzed microarray data obtained using HG-U133A GeneChips (Affymetrix, Santa Clara, CA) to investigate gene expression in xenografts. Raw CEL files were processed using Bioconductor packages in an R environment http://www.bioconductor.org). Quality controls were performed by inspecting Affymetrix^® ^metrics using the simpleaffy package. Probe level signals were then background-corrected, normalized, and summarized using the GC-RMA function, and differential gene expression then computed as above. Click here for file

Additional file 2 **Figure S1**. Metastases in combination SC236 and BV-treated mouse lungs appeared smaller than in control or single-agent treated animals. The low incidence of metastasis in this group (2/15) prevented quantitation of this size reduction, although it may reflect decreased efficiency of metastasis in this group. Click here for file

Additional file 3 **Figure S2**. The addition of SC236 does not alter expression of lung metastasis signature genes during VEGF blockade. We examined expression of 7 genes (COL6A1, CSF2RA, CXCR4, KRT81, MATN2, SPARC, TNC) included in the lung metastasis gene signature described by Minn et al. (8) in tumor extracts from each group (N = 6, controls, SC236-treated, BV-treated; N = 5, BV+SC236 treated). Comparison was performed by real-time PCR. No significant differences between BV and SC236+BV treated tumors were found, suggesting that SC236 did not reduce lung metastasis by suppressing expression of these genes. Click here for file
