# Supplementary figures and images for: Inhibition of cyclo-oxygenase 2 reduces tumor metastasis and inflammatory signaling during blockade of vascular endothelial growth factor
Source: Vasc Cell. 2011 Oct 6;3:22. doi: 10.1186/2045-824X-3-22 (PMC3198683; doi:10.1186/2045-824X-3-22)

Control

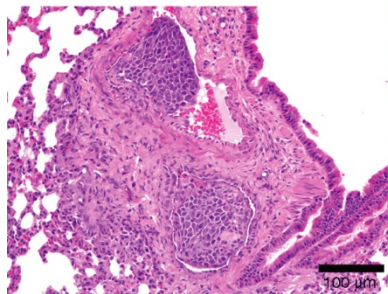

SC236 (10x)

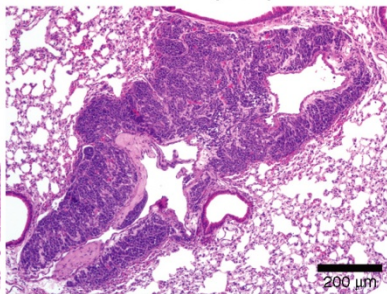

BV

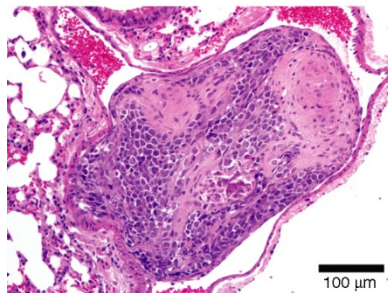

SC236+BV

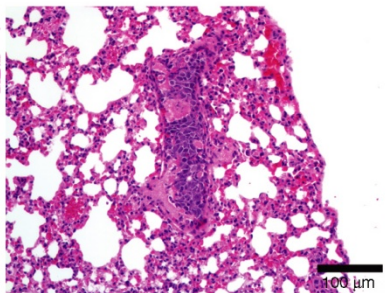

Supplement: Additional file 2 — Figure S1. Metastases in combination SC236 and BV-treated mouse lungs appeared smaller than in control or single-agent treated animals. The low incidence of metastasis in this group (2/15) prevented quantitation of this size reduction, although it may reflect decreased efficiency of metastasis in this group. [file 2045-824X-3-22-S2.PDF]
