# Supplementary material for: Inhibition of cyclo-oxygenase 2 reduces tumor metastasis and inflammatory signaling during blockade of vascular endothelial growth factor
Source: Vasc Cell. 2011 Oct 6;3:22. doi: 10.1186/2045-824X-3-22 (PMC3198683; doi:10.1186/2045-824X-3-22)
Supplement: Additional file 3 — Figure S2. The addition of SC236 does not alter expression of lung metastasis signature genes during VEGF blockade. We examined expression of 7 genes (COL6A1, CSF2RA, CXCR4, KRT81, MATN2, SPARC, TNC) included in the lung metastasis gene signature described by Minn et al. (8) in tumor extracts from each group (N = 6, controls, SC236-treated, BV-treated; N = 5, BV+SC236 treated). Comparison was performed by real-time PCR. No significant differences between BV and SC236+BV treated tumors were found, suggesting that SC236 did not reduce lung metastasis by suppressing expression of these genes. [file 2045-824X-3-22-S3.PDF]

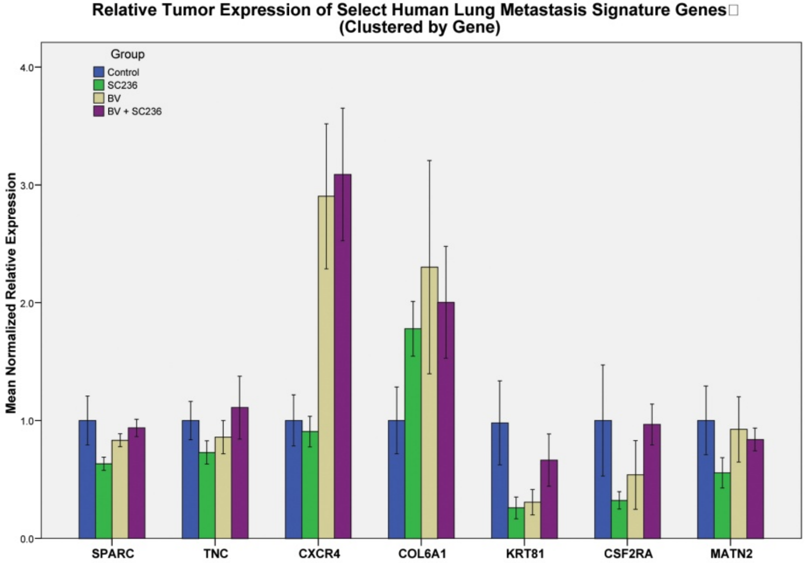

Summary of Statistically Significant Comparisons

Control v SC236:	SPARC	P=0.025 by Kruskal-Wallis	
Control v BV:	CXCR4	P=0.015 by T-test;	P=0.016 by Kruskal-Wallis
Control v BV+SC236:	CXCR4	P=0.012 by T-test;	P=0.004 by Kruskal-Wallis
SC236 v BV:	SPARC	P=0.030 by T-test;	P=0.037 by Kruskal-Wallis
SC236 v BV:	CXCR4	P=0.010 by T-test;	P=0.006 by Kruskal-Wallis
SC236 v BV+SC236:	SPARC	P=0.008 by T-test;	P=0.013 by Kruskal-Wallis
SC236 v BV+SC236:	CXCR4	P=0.011 by T-test;	P=0.004 by Kruskal-Wallis
SC236 v BV+SC236	CSF2RA	P=0.007 by T-test;	P=0.004 by Kruskal-Wallis

No Significant Values Detected for any Gene in BV vs BV+SC236 Comparisons
